# The Impact of Potassium Dynamics on Cardiomyocyte Beating in Hemodialysis Treatment

**DOI:** 10.3390/jcm13082289

**Published:** 2024-04-15

**Authors:** Hiroyuki Hamada, Tadashi Tomo, Sung-Teh Kim, Akihiro C. Yamashita

**Affiliations:** 1 Department of Bioscience and Biotechnology, Faculty of Agriculture, Kyushu University, 744 Motooka, Nishi-ku, Fukuoka-City 819-0395, Japan; hamada@brs.kyushu-u.ac.jp; 2 Clinical Engineering Research Center, Faculty of Medicine, Oita University, 1-1 Idai-Gaoka, Hasama-Machi, Yufu-City 879-5593, Japan; tomo@oita-u.ac.jp; 3 Research Planning Division, Social Medical Corporation Kawashima Hospital, 1-1-39 Kitasako, Tokushima-City 770-0011, Japan; palhae_kim@yahoo.co.jp; 4 Department of Chemical Science and Technology, Faculty of Bioscience and Applied Chemistry, Hosei University, 1-7-2 Kajino-Cho, Koganei-City 184-8584, Japan

**Keywords:** renal replacement therapy, electrophysiology, mathematical analysis, cardiac beating dysfunction, cardiomyocyte, potassium dynamics, excitation–contraction coupling, regulatory science

## Abstract

**Background**: Observational studies of intermittent hemodialysis therapy have reported that the excess decrease in K^+^ concentration in plasma (KP) during treatment is associated with the destabilization of cardiac function. Elucidating the mechanism by which the decrease in KP impairs myocardial excitation is indispensable for a deeper understanding of prescription design. **Methods**: In this study, by using an electrophysiological mathematical model, we investigated the relationship between KP dynamics and cardiomyocyte excitability for the first time. **Results**: The excess decrease in KP during treatment destabilized cardiomyocyte excitability through the following events: (1) a decrease in KP led to the prolongation of the depolarization phase of ventricular cells due to the reduced potassium efflux rate of the Kr channel, temporarily enhancing contraction force; (2) an excess decrease in KP activated the transport of K^+^ and Na^+^ through the funny channel in sinoatrial nodal cells, disrupting automaticity; (3) the excess decrease in KP also resulted in a significant decrease in the resting membrane potential of ventricular cells, causing contractile dysfunction. Avoiding an excess decrease in KP during treatment contributed to the maintenance of cardiomyocyte excitability. **Conclusions**: The results of these mathematical analyses showed that it is necessary to implement personal prescription or optimal control of K^+^ concentration in dialysis fluid based on predialysis KP from the perspective of regulatory science in dialysis treatment.

## 1. Introduction

Many end-stage renal disease patients normally undertake three hemodialysis (HD) treatments per week. Waste products, fluids, and electrolytes that accumulate in the body during non-treatment times are removed from the body at a rapid rate during treatment. It is well known that fluctuations in body fluid properties and circulatory or hemodynamic instability caused by intermittent treatment are associated with dialysis imbalance syndrome and cardiac dysfunction [1,2,3]. Repeated cardiac dysfunction increases patients’ chances of cardiac-related death [2]. The main cause of death in intermittent HD patients is severe cardiovascular complications, and approximately 60% of these patients have a history of cardiac arrest [4]. Therefore, establishing a means to avoid these fatal incidents is essential to achieving a good life prognosis for intermittent HD patients. Regarding hemodynamic instability, Wang et al. reported that the plasma refilling rate at the start of HD and during dialysis is associated with intradialytic hypotension [3]. In this study, we focused on the fluctuations in body fluid properties and investigated the influence of electrolyte dynamics on myocardial excitability during treatment.

DOPPS phase 1, 2, and 3, and USRDS 2015 annual data reports have statistically demonstrated that (1) the large shift in potassium ion concentration in plasma (KP) observed during treatment was associated with cardiac dysfunction, and (2) the risk of sudden cardiac death in patients prescribed high potassium dialysate is lower compared to those prescribed low potassium dialysate [5,6]. These findings implied that excess decrease in KP during treatment should be avoided to maintain cardiac function.

The removal rate of potassium ions (K^+^) in HD treatment is generally larger than that of calcium ions (Ca^2+^) and sodium ions (Na^+^). The K^+^ is transported from the plasma to the dialysate primarily by diffusion, and KP decreases by more than 1.0 mEq/L during treatment. The potassium ion concentration in interstitial fluid (KI) then decreases in conjunction with KP. The Goldman–Hodgkin–Katz equation, which is a fundamental equation in electrophysiology, proved that KI is the predominant factor of the resting membrane potential of excitable cells such as neurons and muscle cells [7]. Therefore, a decrease in KI during treatment of dialysis patients may lower the resting membrane potential and inactivate the excitability of neurons and muscle cells. These insights have prompted clinical researchers to re-evaluate the biocompatibility of the potassium ion concentration in dialysis fluid (KD). DOPPS phase 5 reported that the global mean of KD has increased from 2.0 mEq/L to 2.25 mEq/L in the past few years [8]. Thus, progress is being made in improving the biocompatibility of KD in clinical practice. However, a detailed analysis of the effects of K^+^ dynamics during treatment on the formation of myocardial beating rhythm and the excitation–contraction coupling, based on electrophysiology, has not been conducted. We need to elucidate the mechanism by which a decrease in KP during treatment induces the destabilization of the myocardial beating rhythm and contractile dysfunction and gain a deeper understanding of the essence of prescription design.

The comprehensive in vitro proarrhythmia assay (CiPA) initiative has recommended the application of “in vitro analysis using human induced pluripotent stem cell-derived cardiomyocyte” and “in silico analysis based on electrophysiology of single cardiomyocyte” for the assessment of cardiovascular toxicity of such drugs [9]. We followed the CiPA initiative’s mathematical approach and developed a mathematical analysis system that evaluates the relationship between the fluctuation in electrolyte concentration in body fluids during treatment and cardiomyocyte excitability [10]. In this study, through an in silico approach, we investigated the relationship between K^+^ dynamics and cardiomyocyte beating function during treatment. We also elucidated the detailed mechanisms underlying the destabilization of the beating rhythm in central sinoatrial nodal cells and the decrease in the contraction force of ventricular cells from the viewpoint of electrophysiology. Additionally, we identified cases showing cardiac arrhythmias during treatment and explored therapeutic strategies to stabilize cardiomyocyte beating function. The conclusions of this study advocate strongly for the necessity of implementing personal prescription or optimal control of KD based on predialysis KP, with the aim of avoiding cardiac dysfunction during treatment.

## 2. Materials and Methods

### 2.1. Mathematical Modeling

To evaluate the impact of K^+^ dynamics during chronic HD therapy on cardiomyocyte excitability, a 4-compartment model consisting of dialysis fluid, plasma, interstitial fluid, and intracellular fluid was constructed [10]. Supplementary Figure S1 illustrates the details of this model. The dialysis membrane transport of K^+^ is presented based on the model proposed by Gotch et al. [11]. The K^+^ transfer rate between plasma and interstitial fluid was estimated by employing a transport equation that took into account plasma refilling and distribution. Changes in KI during treatment affected electrolyte transport across the cellular membrane of cardiomyocytes and played a role in disturbing the formation of beating rhythm and the excitation–contraction coupling.

The beating rhythm was evaluated by using a mathematical model representing the excitability of central sinoatrial node cells [10]. In addition, in order to reproduce the fluctuation of interbeat intervals, we ran a stochastic numerical simulation that takes stochasticity into account in the gating of ion channels [12,13,14,15]. In the Supplementary Materials, Figure S2 and Tables S1–S3 provide detailed information about these. On the other hand, in the analysis of contraction, we first performed a deterministic numerical simulation of a mathematical model expressing the excitability of ventricular cells developed by the Rudy Group [16], and we obtained changes in cytosol electrolyte concentration during treatment time. Then, the contraction force was estimated by applying the cytosolic Ca^2+^ concentration to Negroni’s model [17]. These mathematical models of cardiomyocytes reproduced changes in membrane current and membrane potential during treatment based on various ion currents of more than 10 types of ion channels, pumps, and exchange mechanisms, as shown in Table 1. Furthermore, since a detailed elucidation of cardiomyocyte beating function required data about the intracellular local dynamics of electrolytes, the intracellular fluid in the models was divided into the following four spaces: (1) the nearest space to the cellular membrane (subspace); (2) cytosol; (3) the network sarcoplasmic reticulum (NSR); and (4) the junctional sarcoplasmic reticulum (JSR) (Figure S2). Ca^2+^ circulated between the extracellular space and these four compartments, while K^+^ and Na^+^ circulated between the extracellular space, subspace, and cytosol. The CiPA method was utilized to detect the current changes between fluid phases in order to estimate and represent ion concentrations in some fluid compartment, such as intracellular and interstitial spaces.

### 2.2. Mathematical Analysis of Cardiomyocyte Beating

In order to evaluate the impact of predialysis KP on cardiomyocyte pulsation function, the changes in the beating rhythm of central sinoatrial nodal cells and the contraction force of ventricular cells during the treatment of Cases #1 to #3 shown in Table 2 were mathematically analyzed. Table S4 shows patient characteristics and treatment conditions. A patient with a dry weight of 60 kg was treated with HD three times a week for 4 hr per session. The KD was set to 2.0 mEq/L, which is the standard condition in Japan [18]. Predialysis KP was cited from Japanese data from DOPPS phase 5 [8]. The KP for Case #1 corresponds to the 75th percentile, and the predialysis value is high. Cases #2 and #3 are in the 50th and 25th percentiles, respectively. First, we evaluated the effects of weekly time courses of KP and KI on the beating rhythm of central sinoatrial nodal cells and the contraction force of ventricular cells. Next, we explored the mechanisms underlying the destabilization of the beating rhythm and the decrease in contraction force. Finally, we demonstrated that optimizing the KD is effective in avoiding cardiomyocyte beating dysfunction during treatment. The beating rhythm was evaluated by using the average of 50 interbeat intervals, and the significance level for statistical analysis was set at 0.05. The numerical simulation of a 4 hr dialysis treatment required approximately 2 weeks of calculation time (Intel Core i9 (Intel Corporation, Santa Clara, CA, USA); icc compiler (Intel Corporation, Santa Clara, CA, USA)).

## 3. Results

### 3.1. Cardiomyocyte Beating Observed in Case #1

**Figure 1:**
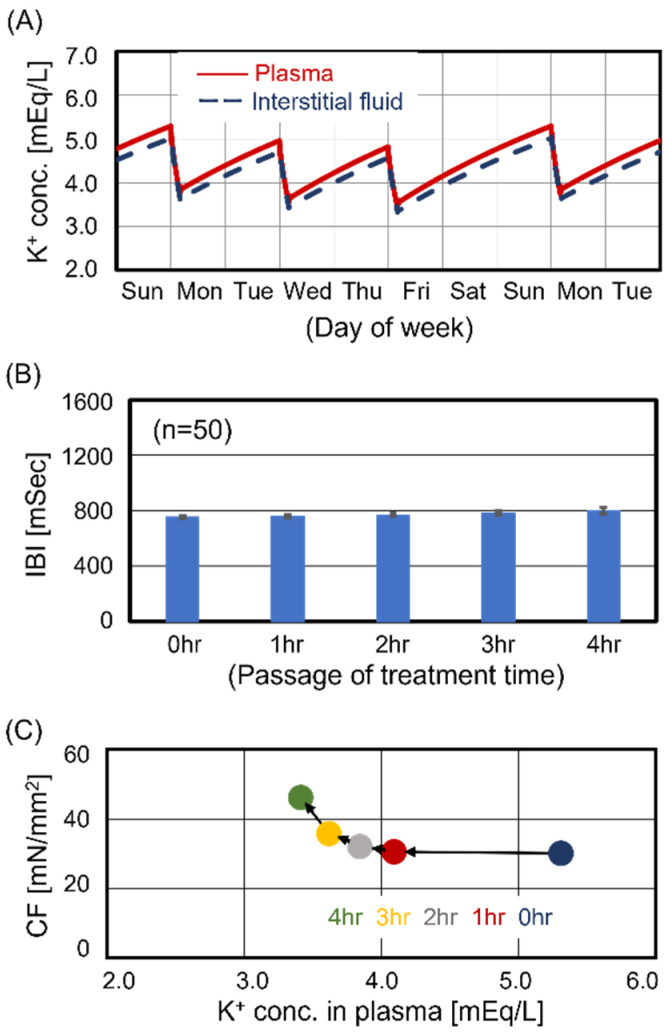
Simulated results for Case #1. Case #1 had no cardiomyocyte dysfunction during the entire treatment time. Panel (**A**): Time courses of potassium concentration in plasma and interstitial fluid. Panel (**B**): Time course of interbeat intervals (IBIs). Panel (**C**): Relationship between contraction force (CF) and K_P_.

Several observational studies have reported that the optimal range for KP is between 4.0 and 5.0 mEq/L [19,20,21]. The predialysis KP for Case #1 was 5.3 mEq/L, indicating concern for hyperkalemia. Figure 1A shows two weekly time courses of KP and KI for Case #1. The KP and KI drastically decreased during HD treatment. Moreover, the transitions showed the oscillatory dynamics for an interval equivalent to 1 week, which implied that the homeostasis balance of the patient was stable, representing the characteristics of maintenance dialysis patients. The changes in KP and KI seen during the first treatment (Monday) in a week were larger than those that occurred during other treatments. Focusing on the Monday treatment, we estimated the beating rhythm of central sinoatrial nodal cells and the contraction force of ventricular cells during treatment. Figure 1B shows the transition of interbeat intervals of central sinoatrial nodal cells during treatment. There were no significant changes in the beating rhythm during treatment. Figure 1C shows the transition of the contraction force of ventricular cells during treatment. While KP decreased with treatment time, contraction force was enhanced. Therefore, Case #1 had no cardiomyocyte beating dysfunction during treatment.

### 3.2. Cardiomyocyte Beating Observed in Case #2

**Figure 2:**
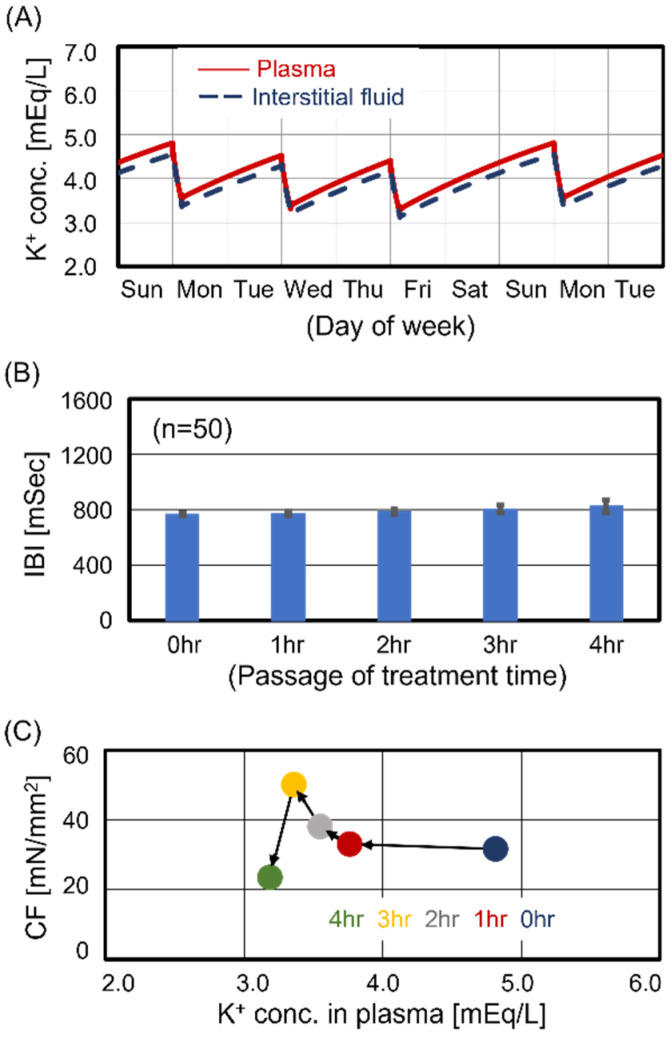
Simulated results for Case #2. Case #2 had no cardiomyocyte dysfunction during the entire treatment time. Panel (**A**): Time courses of potassium concentration in plasma and interstitial fluid. Panel (**B**): Time course of interbeat intervals (IBIs). Panel (**C**): Relationship between contraction force (CF) and K_P_.

The predialysis KP for Case #2 was 4.8 mEq/L, which corresponds to the average value for Japanese HD patients [8]. Figure 2A shows two weekly time courses of KP and KI for Case #2. The weekly transitions of KP and KI were stable, as in Case #1, showing the characteristics of maintenance dialysis patients. Focusing on the Monday treatment, we estimated the beating rhythm of central sinoatrial nodal cells and the contraction force of ventricular cells during treatment. Figure 2B shows the transition of interbeat intervals of central sinoatrial nodal cells during treatment. Similar to Case #1, there were no significant changes in the beating rhythm during treatment. Figure 2C shows the transition of contraction force during treatment. The KP decreased with treatment time. Contraction force was maximized at 3 hr after the start of treatment and slightly decreased during the last hour. Thus, although the contraction force for Case #2 slightly decreased during the last hour of treatment, the cardiomyocyte beating was maintained during treatment.

### 3.3. Cardiomyocyte Beating Observed in Case #3

**Figure 3:**
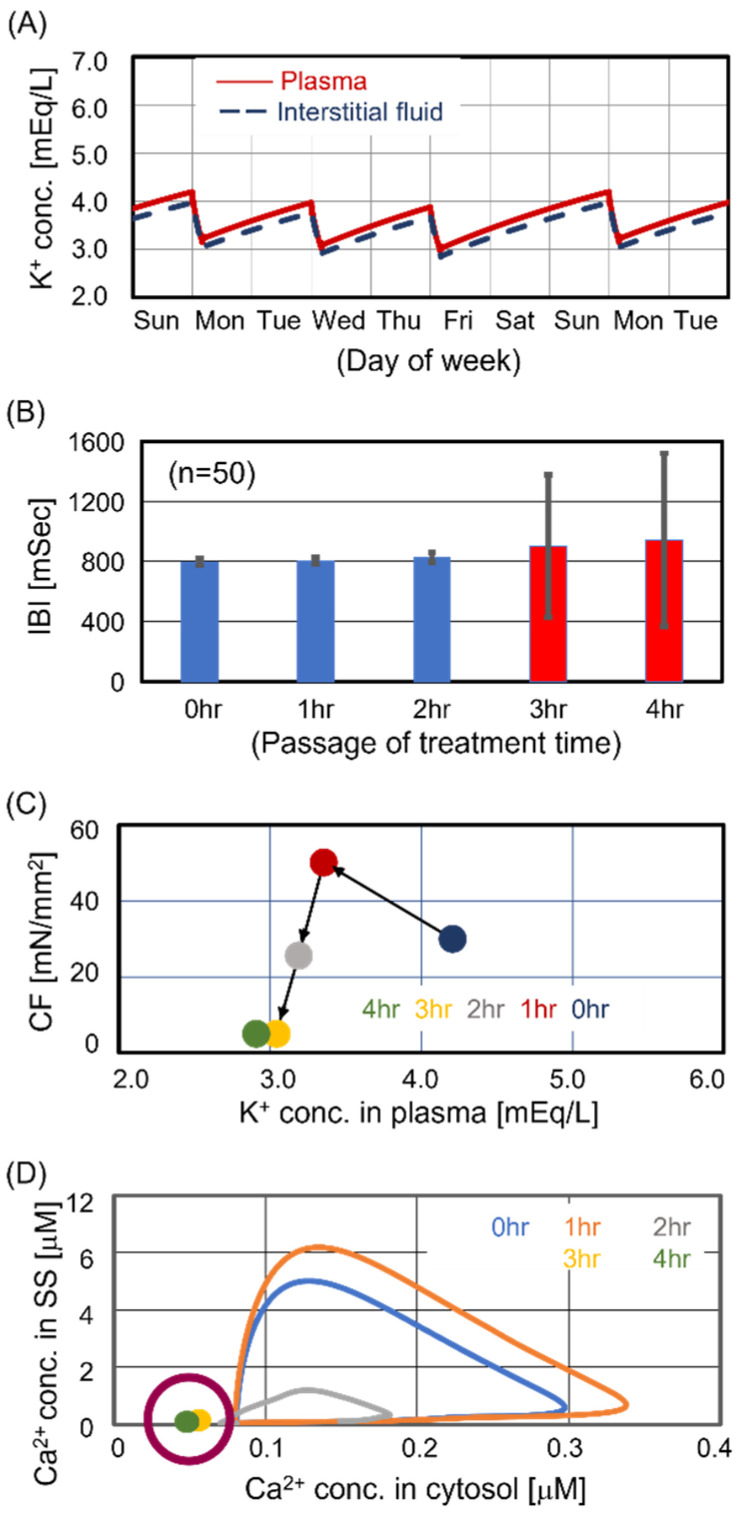
Simulated results for Case #3. Case #3 showed severe cardiomyocyte dysfunction later in the treatment. Panel (**A**): Time courses of potassium concentration in plasma and interstitial fluid. Panel (**B**): Time course of interbeat intervals (IBIs). The IBI for 3 hr and 4 hr showed a significant difference from that for 0 h (*p* < 0.05). Panel (**C**): Relationship between contraction force (CF) and K_P_. Panel (**D**): Relationship between calcium concentration in subspace (SS) and calcium concentration in the cytosol. The Ca^2+^ cycling at 3 h and 4 h within the magenta circle was arrested, indicating contractile dysfunction.

The predialysis KP for Case #3 was 4.2 mEq/L, which was within the optimal range based on observational studies of KP [19,20,21]. Figure 3A shows two weekly time courses of KP and KI. While the weekly transitions of KP and KI were stable, as in Cases #1 and #2, their amplitudes decreased. Focusing on the Monday treatment, we estimated the beating rhythm of central sinoatrial nodal cells and the contraction force of ventricular cells during treatment. Figure 3B shows the transition of interbeat intervals during treatment. The beating rhythm was significantly disrupted in the last half of the treatment. This study captured the unstable beating rhythm during HD treatment for the first time through mathematical analysis. Figure 3C shows the transition of contraction force during treatment. The KP decreased with treatment time. Contraction force was maximized at 1 hr after the start of treatment and significantly decreased during the last half of the treatment. To evaluate the activity of the excitation–contraction coupling in the last half of the treatment, we analyzed the intracellular Ca^2+^ cycling dynamics, which is the dominant factor in the contraction mechanism [22]. Figure 3D shows the relationship between the Ca^2+^ concentration in the subspace and the Ca^2+^ concentration in the cytosol. In this figure, the clockwise closed curve, called a limit cycle, indicates the intracellular Ca^2+^ cycling dynamics. Additionally, the area of the limit cycle correlates with the intensity of the contraction force. The Ca^2+^ cycling was arrested at 3 and 4 hr after the start of treatment, indicating contractile dysfunction. In summary, cardiomyocytes in Case #3 maximized contraction force at 1 hr after the start of treatment but showed cardiomyocyte beating dysfunction in the last half of the treatment.

### 3.4. Unstable Beating Rhythm

**Figure 4:**
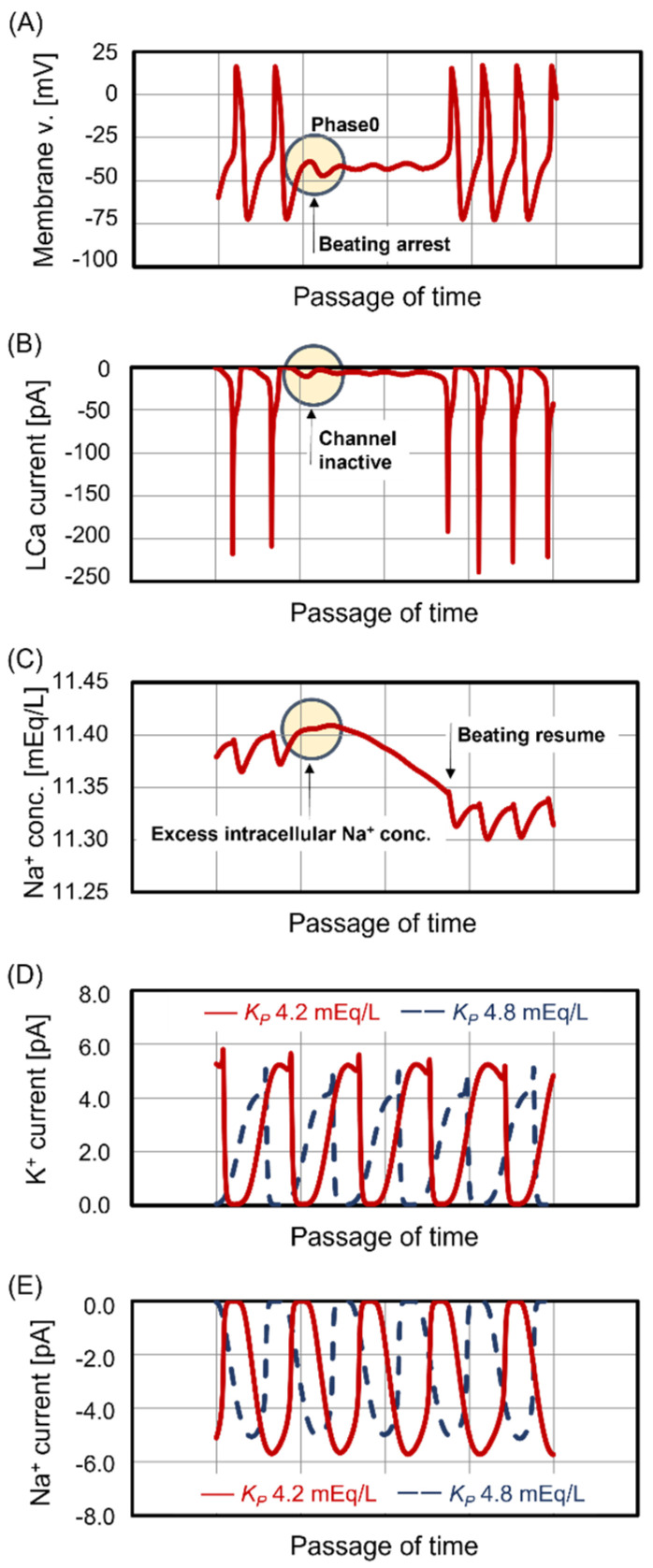
Disruption of beating function in sinoatrial nodal cells. Activation of ion transport through funny channel led to disruption of beating function in sinoatrial nodal cells. Panel (**A**): Time course of membrane voltage when beating was arrested. Panel (**B**): Time course of Ca^2+^ current of L-type Ca channel (LCa current). Panel (**C**): Time course of Na^+^ concentration in cytosol. Panel (**D**): Time course of K^+^ current of funny channel. Panel (**E**): Time course of Na^+^ current of funny channel.

Case #3 exhibited an unstable beating rhythm in the last half of the treatment, as shown in Figure 3B. Figure 4A shows the temporal changes in the membrane potential of central sinoatrial nodal cells when the unstable beating rhythm was observed. The transitions in membrane potential were arrested during the depolarization phase (phase 0), which implied the inactivation of membrane potential-dependent ion channels that transport Na^+^ and Ca^2+^ into the cells. Figure 4B shows the ion current of L-type Ca^2+^ channels, which dominate the influx of Ca^2+^ during phase 0. The L-type Ca^2+^ channels remained closed, and no influx of Ca^2+^ into the cells was observed. Figure 4C shows the temporal changes in intracellular Na^+^ concentration. The intracellular Na^+^ concentration increased with treatment time and reached a peak when the beating rhythm became unstable. Subsequently, central sinoatrial nodal cells paused beating and decreased the intracellular Na^+^ concentration through functions such as the Na/K pump. When the intracellular Na^+^ concentration slightly decreased, central sinoatrial nodal cells resumed beating. However, as the decrease was insufficient, the intracellular Na^+^ concentration reached a peak again, leading to a pause of beating. This was a repeatable event. To explore the reason for the increase in intracellular Na^+^ concentration during treatment, all ion currents considered in the mathematical model of central sinoatrial nodal cells were comprehensively examined. The results revealed significant changes in the funny channel ion current. The funny channel transports K^+^ out of the cells and Na^+^ into the cells during the diastolic depolarization phase (phase 4), generating pacemaker currents in the central sinoatrial nodal cells [23]. Figure 4D shows the temporal changes in the K^+^ current of the funny channel. The area under the curve of K^+^ current for Case #3 (red curve) was larger than that for Case #2 (blue curve), which implied that the transport amount of K^+^ in Case #3 is larger than that in Case #2. Figure 4E shows the temporal changes in the Na^+^ current of the funny channel. The area over the curve of Na^+^ current for Case #3 (red curve) was larger than that for Case #2 (blue curve), which implied that the transport amount of Na^+^ in Case #3 is larger than that in Case #2. In summary, the excess decrease in KP observed during treatment in Case #3 (Figure 3A) increased the extracellular K^+^ efflux and intracellular Na^+^ influx through the funny channel (Figure 4D,E), resulting in the elevation of intracellular Na^+^ concentration during treatment, as shown in Figure 4C, and a pause of beating. This mechanism underlies the instability of the beating rhythm of central sinoatrial nodal cells (Figure 3B).

### 3.5. Transition of Contraction Force

**Figure 5:**
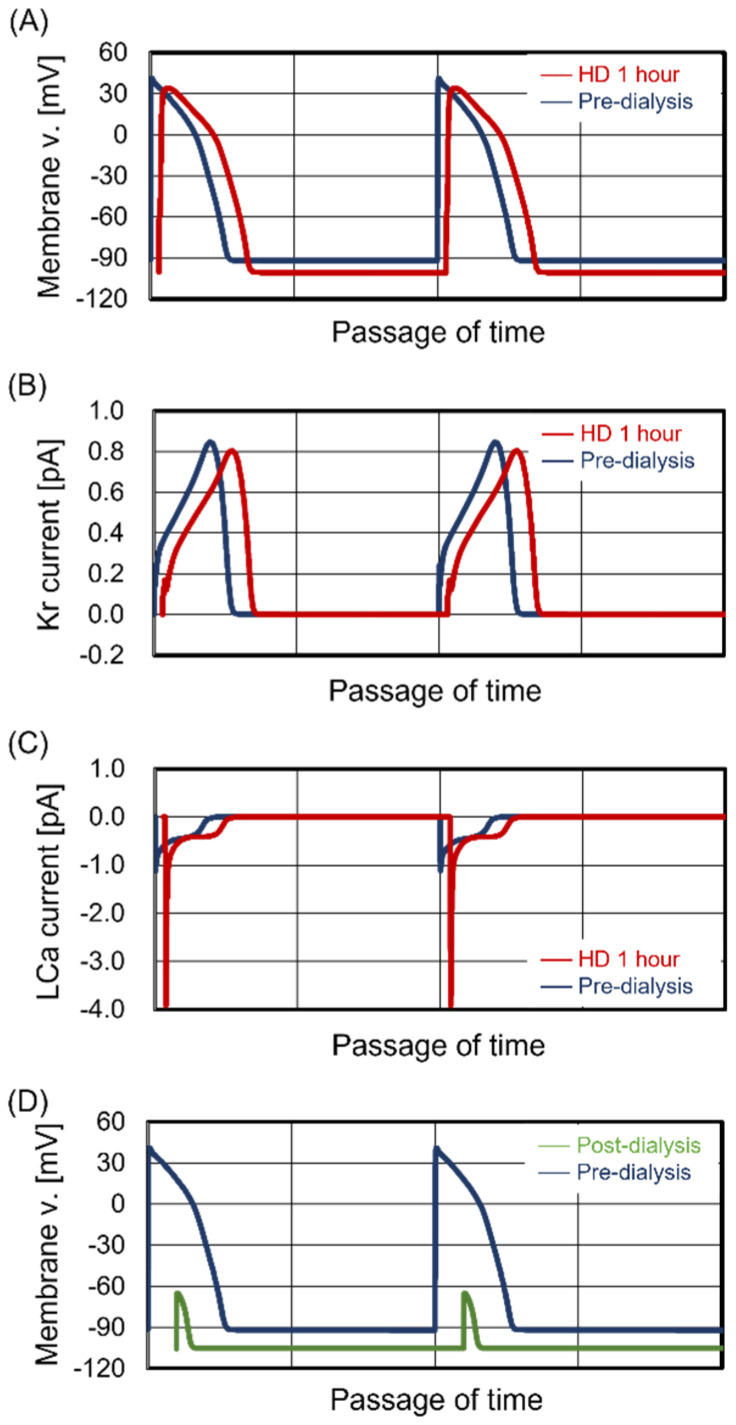
Effect of K^+^ dynamics on the ion transporters in ventricular cells. Slight decrease in K_P_ elongated the depolarization phase, which induced the temporal upregulation of the contraction force as shown in Figure 3C. In contrast, an excess decrease leads to pulsatile insufficiency. Panel (**A**): Decrease in resting membrane potential at 1 hr after starting treatment (HD 1 hour). Panel (**B**): Decrease in K^+^ outward current of the Kr channel at HD 1 hr. Panel (**C**): Increase in Ca^2+^ inward current of the L-type Ca channel at HD 1 hr. Panel (**D**): Pulsatile insufficiency due to excess decrease in K_P_.

Case #3 exhibited the peak of contraction force at 1 hr after the start of treatment, as shown in Figure 3C. Figure 5A shows a comparison of the time course of membrane potential for ventricular cells before treatment (blue curve) with that at 1 hr after the start of treatment (red curve). The resting membrane potential at 1 hr after the start of treatment was lower than that before treatment. Figure 5B shows the K^+^ current of the Kr channel which dominates K^+^ efflux during the depolarization phase. The K^+^ current at 1 hr after the start of treatment (red curve) decreased compared to that before treatment (blue curve), which implied a reduction in the K^+^ efflux rate. This reduction slowed down the rate of decrease in membrane potential, inducing the prolongation of the depolarization phase observed in Figure 5A (red curve). Furthermore, this prolongation increased the uptake of Ca^2+^ through L-type Ca^2+^ channels (Figure 5C) and activated Ca^2+^-induced Ca^2+^ release [24], enhancing contraction force. On the other hand, Case #3 showed a decrease in contraction force in the last half of treatment, as seen in Figure 3C. Figure 5D shows a comparison of the time course of membrane potential for ventricular cells before treatment (blue curve) with that at the end of treatment (green curve). The excess decrease in KP drastically lowered the resting membrane potential, inhibiting the depolarization of ventricular cells. As a result, excitation–contraction coupling was not activated, and contractile dysfunction was observed.

### 3.6. Stabilization of Cardiomyocyte Beating in Case #3

**Figure 6:**
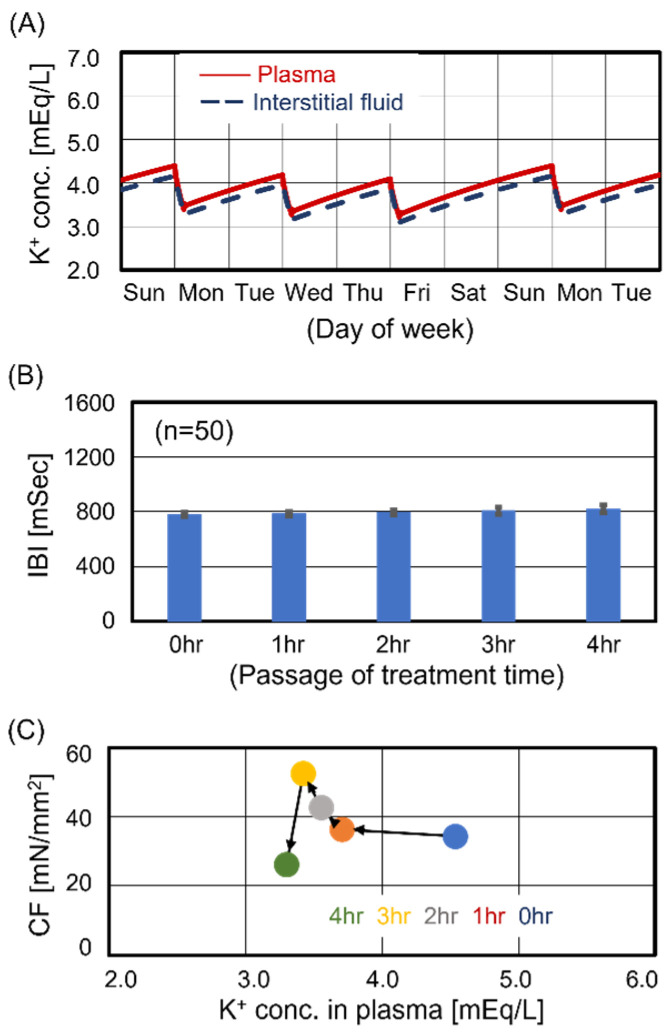
Simulated results for Case #3 used a K_D_ of 2.3 mEq/L. Case #3 avoided cardiomyocyte dysfunction by employing a K_D_ of 2.3 mEq/L. Panel (**A**): Time courses of potassium concentration in plasma and interstitial fluid. Panel (**B**): Time course of interbeat intervals (IBIs). Panel (**C**): Relationship between contraction force (CF) and K_P_.

Cases #1 and #2 maintained cardiomyocyte beating function during treatment, as shown in Figure 1 and Figure 2. However, Case #3 experienced an excess decrease in KP during treatment, leading to an unstable beating rhythm of central sinoatrial nodal cells and contractile dysfunction of ventricular cells during treatment, as shown in Figure 4 and Figure 5. These findings demonstrate that avoidance of excess decrease in KP is essential for maintaining cardiomyocyte beating function during treatment of Case #3. These results were in good agreement with summaries from DOPPS phases 1, 2, and 3, and USRDS 2015 annual data reports [5,6]. Figure 6A shows two weekly time courses of KP and KI when applying a KD of 2.3 mEq/L to Case #3. The weekly variations in KP and KI became similar to those of Case #2. Focusing on the Monday treatment, we estimated the beating rhythm of central sinoatrial nodal cells and the contraction force of ventricular cells during treatment. Figure 6B presents the transition of interbeat intervals during treatment, showing no disruption in the beating rhythm during treatment. Figure 6C shows the transition of the contraction force during treatment. The KP decreased with treatment time. Similar to Case #2, contraction force was maximized at 3 hr after the start of treatment and slightly decreased during the last hour. In summary, applying a KD of 2.3 mEq/L to Case #3 increased the weekly time courses of KP and KI, stabilized the beating rhythm of central sinoatrial nodal cells, and slightly weakened the contraction force of ventricular cells. Consequently, Case #3 maintained cardiomyocyte beating function during treatment. In clinical practice, saline solutions tend to be administered palliatively to cases that show unstable cardiac beating function in the last half of treatment. While this intervention may be effective at improving hemodynamics, it may be ineffective at improving electrolyte dynamics. The simulated results shown in Figure 6 highlight the importance of appropriate potassium administration in cases where electrolyte dynamics fluctuate significantly during HD treatment.

## 4. Discussion

The effects of deviations from normal serum K^+^ levels have been studied in detail in both physiological and pathophysiological settings [25,26]. In the general population, there is greater interest in hyperkalemia than hypokalemia. However, observational studies in chronic HD patients have reported that the risk of mortality associated with hypokalemia may be higher than that associated with hyperkalemia [19,27,28,29]. Nevertheless, to date, there has been little electrophysiology-based basic research on the relationship between excess decrease in KP due to intermittent HD treatment and cardiac beating function. Understanding the impact of excess decrease in KP during treatment on cardiomyocyte beating function is essential for achieving favorable patient outcomes. In this study, based on the concept of CiPA, the dynamic characteristics of cardiomyocyte beating function in intermittent HD patients were examined by using electrophysiological mathematical analysis. The research findings are highly innovative and contribute significantly to the development of renal replacement therapy by elucidating the relationship between KP dynamics and cardiomyocyte beating dysfunction.

Cases #1, #2, and #3, as shown in Table 1, correspond to high, average, and low predialysis KP, respectively. We evaluated the effects of KP dynamics during treatment on the beating rhythm of central sinoatrial nodal cells and the contraction force of ventricular cells in these cases. While Cases #1 and #2 exhibited no cardiomyocyte beating dysfunction during treatment, Case #3 showed a significant decrease in contraction force and destabilization of the beating rhythm during the last half of treatment, as shown in Figure 3. The minimum KP level in Case #3 was lower than that in Cases #1 and #2. These simulated results were consistent with clinical observations from DOPPS phases 1, 2, and 3, and USRDS 2015 annual data reports [5,6], which suggested that an excess decrease in KP may induce cardiac dysfunction during treatment. Our in silico study captured the essence of the dynamic characteristics of cardiac function during dialysis treatment.

**Figure 7:**
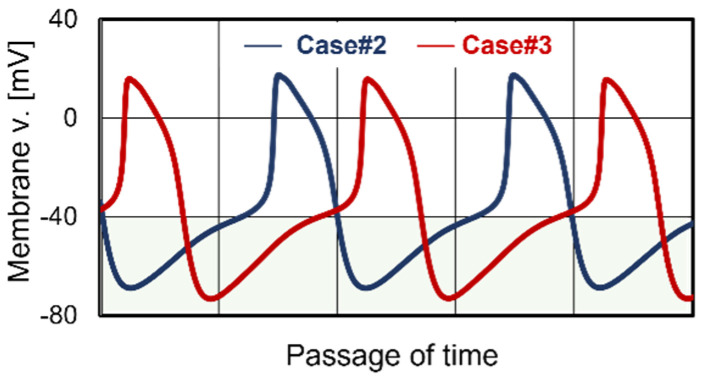
Comparison of time courses of membrane voltages for sinoatrial nodal cells. The blue curve is the profile for Case #2, and the red curve is the profile for Case #3. Electrolyte transfer of the funny channel is activated in the diastolic depolarization phase represented by green color. The minimum membrane voltage in the red curve was around 4 mV lower than that in the blue curve. Therefore, the number of transported electrolytes through the funny channel in the red curve is larger than that in the blue curve.

In the analysis of Case #3, an excess decrease in KP during treatment led to the destabilization of the beating rhythm of the central sinoatrial nodal cells (Figure 3B). Two factors contributed to this destabilization. Firstly, the decrease in KP lowered KI and increased the transport amount of intracellular K^+^ and extracellular Na^+^ through the funny channel (Figure 4D,E). Additionally, the decrease in KP lowered the minimum membrane potential of the central sinoatrial nodal cells. Figure 7 shows a comparison of the time course of membrane potential for Case #3 with that for Case #2. The diastolic depolarization phase in Case #3 was longer than that in Case #2, extending the active period of the funny channel per beat. Consequently, the transport amount of K+ and Na+ through the funny channel in Case #3 was much larger than that in Case #2. These mechanisms led to an excess increase in intracellular Na^+^ concentration and a pause of beating, resulting in destabilization of the beating rhythm. Ivabradine, which is metabolized by the liver, is an inhibitor of the funny channel and elongates the diastolic depolarization phase of the action potential [30]. The application of Ivabradine to cases with low predialysis KP, such as Case #3, may inhibit the excess transport of K^+^ and Na^+^ during treatment, contributing to the stabilization of the beating rhythm. While it is necessary to examine the half-life of Ivabradine concentration during dialysis treatment, such drugs are anticipated as stabilizers of the beating rhythm.

**Figure 8:**
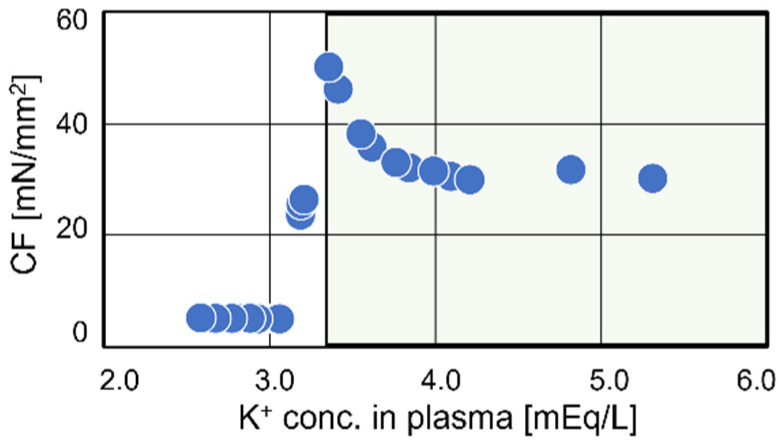
The relationship between contraction force and K_P_. The excess decrease in K_P_ induced cardiomyocyte dysfunction. Our mathematical analysis showed that K_P_ over the entire treatment time should be over 3.4 mEq/L.

In Case #3, transient enhancement of the contraction force was observed during the first half of treatment (Figure 5). El-Sherif., White et al., and Pogwizd et al. reported that slightly decreased extracellular K^+^ concentration prolonged the depolarization phase in both guinea pig and rabbit heart preparations [31,32,33]. Additionally, Beatriz et al. demonstrated that a slight decrease in extracellular K^+^ concentration blunted the potassium efflux rates of the K1, Kr, and Ks channels during the depolarization phase, leading to an extension of the depolarization duration [34]. In particular, we identified the Kr channel, which transports the highest amount of K^+^, as the dominant factor for the enhancement of contraction force (Figure 5B). Figure 8 shows the relationship between KP and contraction force. Our mathematical analysis revealed that contraction force was maximized at a KP level of 3.4 mEq/L and drastically decreased below 3.3 mEq/L. This finding implies that the KP at the end of treatment should be maintained above 3.4 mEq/L. The Kidney Disease: Improving Global Outcomes (KDIGO) Controversies Conference reported interventions for KP levels below 3.5 mEq/L [35]. Our simulated results are consistent with these biological observations and support the findings of the KDIGO Controversies Conference.

Based on the analysis of Case #3, the progression of cardiomyocyte beating dysfunction during treatment is as follows: (1) the contraction force of ventricular cells is decreased; (2) the beating rhythm of central sinoatrial nodal cells is destabilized; and (3) the cardiomyocyte beating function is arrested. When compared to the dynamics of cardiac function during dialysis treatment, these correspond to a series of events leading to the occurrence of hypotension, induction of arrhythmia, and cardiac arrest. These findings suggest a potential association between K^+^ dynamics and the occurrence of hypotension during treatment. Consensus definitions for hypokalemia were not obtained at the Kidney Disease: Improving Global Outcomes (KDIGO) Controversies Conference [35]. In future studies, it is crucial to identify the plasma K^+^ level at which contraction force is maximized (KP_CFP). Figure 6, which shows the effect of KD on cardiomyocyte beating function, indicates that the KP at the end of treatment approximately reaches KP_CFP, thereby avoiding cardiomyocyte beating dysfunction during treatment. Thus, treatment strategies that optimize KD to ensure that the KP at the end of treatment reaches KP_CFP represent an effective means of avoiding cardiomyocyte beating dysfunction during treatment. Optimal control of KD to maintain a constant gradient of between KP and KD [36] may contribute to avoiding excess increases in intracellular Na^+^ concentration in central sinoatrial nodal cells (Figure 4C), thereby becoming a favorable therapeutic strategy for cardiac function. The development of techniques for optimizing or controlling KD based on predialysis KP is essential from the perspective of regulatory science for intermittent HD therapy.

In this paper, we focused on three representative cases and thoroughly examined the impact of potassium ion dynamics during dialysis therapy on cardiomyocyte excitability. In a future, we plan to shift our focus to the group of cases with low predialysis KP highlighted in this paper, gather cases, and conduct a more detailed investigation into the effects of potassium ion dynamics during dialysis therapy on cardiomyocyte excitability. We also aim to propose recommended values for the optimal KD at start of treatment based on KP. Furthermore, we intend to reflect these results in the optimal control of KD. Finally, we apply artificial intelligence to the collected cases and conduct regression analysis to develop a system that outputs the appropriate value for KD at the start of treatment. This system will greatly contribute to the advancement of home hemodialysis.

## 5. Conclusions

The relationship between K^+^ dynamics during intermittent HD treatment and cardiomyocyte beating function was examined using an electrophysiological mathematical model. The decrease in KP during treatment destabilized the beating rhythm of central sinoatrial nodal cells and the excitation–contraction coupling of ventricular cells. The progression of this destabilization was as follows: (1) decreased KP prolonged the depolarization phase via reduced K^+^ efflux rates of Kr channels in ventricular cells, thereby enhancing contraction force; (2) an excess decrease in KP activated the transport of K^+^ and Na^+^ through the funny channel in central sinoatrial nodal cells, inducing the instability of the beating rhythm of these cells; and (3) the excess decrease in KP also drastically lowered the resting membrane potential, inhibiting the depolarization of ventricular cells. Avoidance of an excess decrease in KP during treatment contributed to the maintenance of myocardial beating function. The results of this study show that it is necessary to strongly promote the optimization or optimal control of KD based on predialysis KP from the perspective of regulatory science in dialysis treatment.

## Figures and Tables

**Table 1:** Ion transporters in mathematical models.

Central SA Nodal Cell	Ventricular Cell	Transport Mechanism
Funny ch. (Na^+^, K^+^)	Fast Na ch. (Na^+^)	Voltage-dependent
L-type Ca ch. (Ca^2+^)	Late Na ch. (Na^+^)	Voltage-dependent
T-type Ca ch. (Ca^2+^)	L-type Ca ch. (Ca^2+^)	Voltage-dependent
Ks ch. (K^+^)	Ks ch. (K^+^)	Voltage-dependent
Kr ch. (K^+^)	Kr ch. (K^+^)	Voltage-dependent
Sus ch. (K^+^)	to ch. (K^+^)	Voltage-dependent
4Ap ch. (K^+^)	K1 ch. (K^+^)	Voltage-dependent
Na/Ca exchanger (Na^+^, Ca^2+^)	Na/Ca exchanger (Na^+^, Ca^2+^)	Ca^2+^ conc.-dependent
Na/K pump (Na^+^, K^+^)	Na/K pump (Na^+^, K^+^)	Constitutive
Ca pump (Ca^2+^)	Ca pump (Ca^2+^)	Constitutive
Ryanodine receptor (Ca^2+^)	Ryanodine receptor (Ca^2+^)	Ca^2+^ conc.-dependent
SERCA pump (Ca^2+^)	SERCA pump (Ca^2+^)	Constitutive
Leak (Na^+^, Ca^2+^)	Leak (Na^+^, Ca^2+^)	Constitutive

Transported ion is shown in parentheses.

**Table 2:** Treatment conditions for 3 cases of interest.

Case #	Electrolytes Concentration in Dialysis Fluid [mEq/L]			Predialysis Electrolytes Concentration in Plasma [mEq/L]			Japanese Data in DOPPS Phase 5 *
	Ca^2+, $^	Na^+, $^	K^+, $^	Ca^2+, $^	Na^+, $^	K^+, $^	Percentile
1	2.75	140.0	2.0	2.5	140.0	5.2 *	75 (High)
2						4.8 *	50 (Average)
3						4.2 *	25 (Low)

^$^ Japanese typical data [18]. * Japanese data in DOPPS Phase 5 [8].

## Data Availability

The original contributions presented in the study are included in the article/Supplementary Materials, further inquiries can be directed to the corresponding author.

## Supplementary Materials

The following supporting information can be downloaded at https://www.mdpi.com/article/10.3390/jcm13082289/s1. Figure S1. Schematic drawing of the four-compartment model; Figure S2. Schematic drawing of a sinoatrial nodal cell; Table S1. Initial conditions for mathematical model of sinoatrial nodal cells; Table S2. Constants for mathematical model of sinoatrial nodal cells; Table S3. Mathematical equations of sinoatrial nodal cells; Table S4. Patient physical properties and treatment conditions.
