# Supplementary material for: The Impact of Potassium Dynamics on Cardiomyocyte Beating in Hemodialysis Treatment
Source: J Clin Med. 2024 Apr 15;13(8):2289. doi: 10.3390/jcm13082289 (PMC11051548; doi:10.3390/jcm13082289)
Supplement: Supplementary file 1 [file jcm-13-02289-s001.zip › Supplementary Materials--- Figures.pdf]

Supplementary Materials

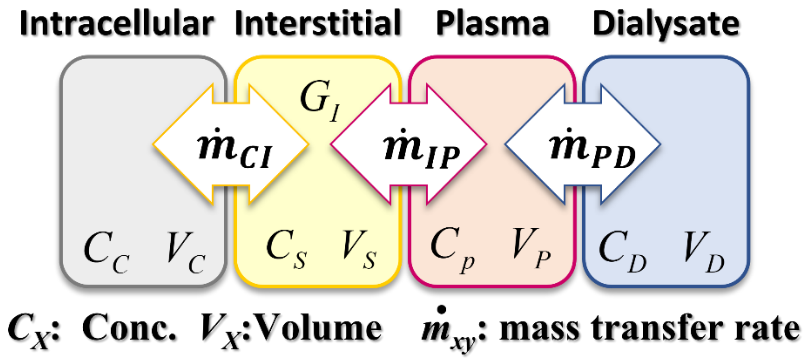

Figure S1. Schematic drawing of the 4-compartment model

ICF, IF, Plasma and DF = intracellular fluid, interstitial fluid, plasma and dialysis fluid, respectively. Mass transfer rates between those spaces are the $\dot{m}_{CI}$, $\dot{m}_{IP}$ and $\dot{m}_{PD}$. The electrolyte concentration and volume in each space are C_C and V_C for ICF, C_I and V_I for IF, C_P and V_P for Plasma, and C_D and V_D for DF.

The rights in the material are owned by a third party [10].

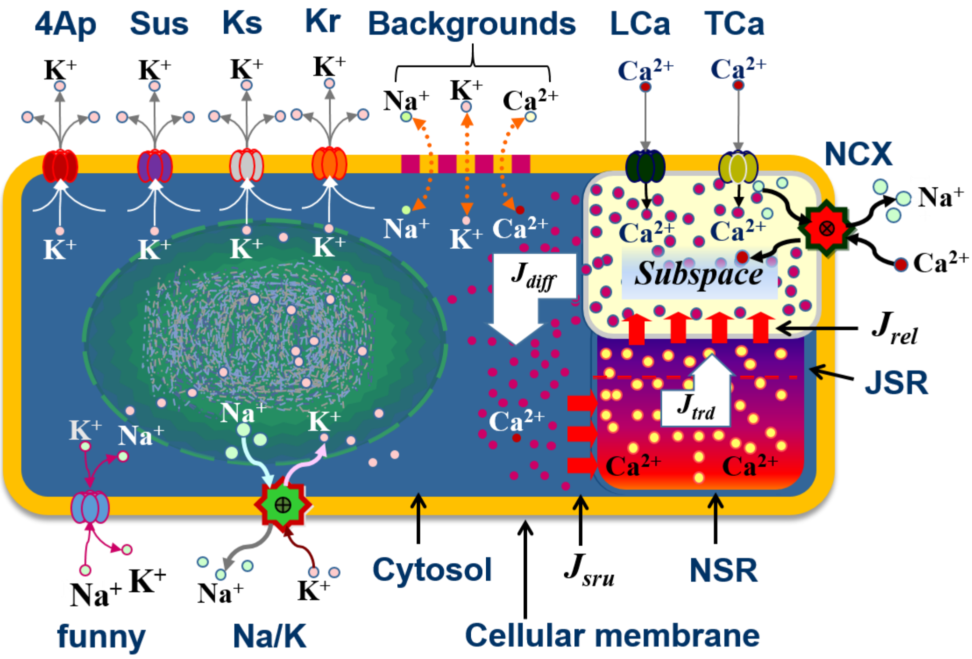

Figure S2. Schematic drawing of sinoatrial nodal cell

Proposed model represented the time courses for membrane voltage (V_m) and membrane current (I_{tot}) changes, based on the transition of each ion current in more than 10 types of ion channel, the pumps, and the exchanger. Moreover, since a detailed elucidation of excitation-contraction coupling required data about the intracellular local dynamics of electrolytes, the intracellular fluid in the models was divided into the following four spaces: (1) the nearest space to the cellular membrane (subspace); (2) cytosol; (3) the network sarcoplasmic reticulum (NSR); and (4) the junctional sarcoplasmic reticulum (JSR). With each cardiomyocyte beat, Ca^{2+} was released from the JSR to the subspace and transported to the JSR via the cytosol and NSR (Ca^{2+} circulation). Thus, Ca^{2+} concentration in subspace was corresponded to C_C in Fig. 1. In contrast, Na^+ and K^+ were transferred between the extracellular space and the cytosol. Hence, Na^+ and K^+ concentrations in cytosol were corresponded to C_C in Fig. 1. Transporters were abbreviated as follows: LCa = L-type Ca^{2+} channel, TCa = T-type Ca^{2+} channel, Kr = Kr channel, Ks = Ks channel, 4Ap = 4-aminopyridine-sensitive ion channel, funny = funny channel, Sus = Sustained ion channel, NCX = Na/Ca exchanger, Na/K = Na/K pump, and Backgrounds = Na^+ K^+, Ca^{2+} background. The Ca^{2+} fluxes were abbreviated as follows: J_{rel} = ryanodine receptors Ca^{2+} release flux, J_{diff} = Ca^{2+} transfer rate from subspace to cytosol, J_{sru} = SERCa pump Ca^{2+} uptake flux from cytosol to NSR, and J_{trd} = Ca^{2+} transfer rate from NSR to JSR. Table S3 shows mathematical equations of ion current in transporters and kinetic models for Ca^{2+} fluxes.

The rights in the material are owned by a third party [10].

[10]. Hamada, H.; Tomo, T.; Kim, ST.; Hanai, T.; Okamoto, M.; Yamashita, A.C. Electrophysiological insights into the relationship between calcium dynamics and cardiomyocyte beating function in chronic hemodialysis treatment. *J. Artif. Organs.* **2021**, *24*, 58–64.
