# Supplementary material for: The Impact of Potassium Dynamics on Cardiomyocyte Beating in Hemodialysis Treatment
Source: J Clin Med. 2024 Apr 15;13(8):2289. doi: 10.3390/jcm13082289 (PMC11051548; doi:10.3390/jcm13082289)
Supplement: Supplementary file 1 [file jcm-13-02289-s001.zip › Supplementary Materials--- Tables.pdf]

Supplementary Materials

Table S1. Initial conditions for mathematical model of sinoatrial nodal cells

K^+ concentration in interstitial fluid (K_I)	4.8 mM [15]
K^+ concentration in cytosol (K_C)	140.0 mM [15]
Na^+ concentration in interstitial fluid (Na_I)	140.0 mM [15]
Na^+ concentration in cytosol (Na_C)	10.0 mM [15]
Ca^{2+} concentration in plasma (Ca_P)	1.150 mM
Ca^{2+} concentration in interstitial fluid (Ca_I)	1.085 mM
Ca^{2+} concentration in cytosol (Ca_C)	0.0001 mM [15]
Ca^{2+} concentration in subspace (Ca_S)	0.000223 mM [15]
Ca^{2+} concentration in junctional sarcoplasmic reticulum (JSR)	0.029 mM [15]
Ca^{2+} concentration in network sarcoplasmic reticulum (NSR)	1.35 mM [15]
Membrane voltage	-65.0 mV
Gate opening stochasticity	
Funny channel y (active state)	1.0
L type Ca channel dL (active state)	0.0
L type Ca channel fL (inactive state)	1.0
L type Ca channel fC(inactive state)	0.0
T type Ca channel dT (active state)	0.0
T type Ca channel fT(inactive state)	1.0
Ks channel n (active state)	0.0
Kr channel PaF (active state)	0.0
Kr channel PaS (active state)	0.0
Kr channel Pi (inactive state)	1.0
Sustained qa (active state)	0.0
Sustained qi (inactive state)	1.0
4AP channel q (inactive state)	1.0
4AP channel r (active state)	0.0
ryanodine receptor R (closed state)	0.75
ryanodine receptor I (inactive state)	0.11E-5
ryanodine receptor O (open state)	0.34E-6
ryanodine receptor RI (refractory state)	0.25
Ca^{2+} buffering	
Troponin_Ca (for Ca^{2+} binding) (TC_{tot})	0.031 mM [15]
Fraction of Toponin_Ca and Ca^{2+} complex in cytosol (*fTC*)	2.0 %
Troponin_Mg (for Mg^{2+} binding) (TMC_{tot})	0.062 mM [15]
Fraction of Toponin_Mg and Ca^{2+} complex in cytosol (*fTMC*)	22.0 %
Fraction of Toponin_Mg and Mg^{2+} complex in cytosol (*fTMM*)	69.0 %
Calsequestrin (CQ_{tot})	10.0 mM [13]
Fraction of Calsequestirn and Ca^{2+} complex in JSR (*fCQ*)	22.0 %
Calmodulin (CM_{tot})	0.045 mM [13]
Fraction of Calmodulin and Ca^{2+} complex in cytosol (*fCMi*)	4.2 %
Fraction of Calmodulin and Ca^{2+} complex in subspace (*fCMs*)	8.9 %

Table S2. Constants for mathematical model of sinoatrial nodal cells

Cellular electric capacitance (C_m)	32.0 pF [15]
Faraday constant (F)	96485.0 C/mol [15]
Cell volume (V_{cell})	3.518 pL [13]
Subspace volume (V_{sub})	0.035 pL [13]
Cytosol volume (V_{cyt})	1.583 pL [13]
Network sarcoplasmic reticulum volume (V_{NSR})	0.041 pL [13]
Junctional sarcoplasmic reticulum volume (V_{JSR})	0.004 pL [13]
Mg^{2+} concentration in cytosol	2.5 mM [15]
Single channel conductance (siemens)	
funny channel	0.5 pS [14]
L type Ca channel	3.6 pS [14]
T type Ca channel	5.8 pS [14]
K channel	2.9 pS [14]
Whole cell conductance (siemens)	
funny channel	4.8 nS [15]
L type Ca channel	18.56 nS [13]
T type Ca channel	5.86 nS [15]
Ks channel	0.83 nS [13]
Kr channel	2.60 nS [15]
Sustained channel	0.096 nS [15]
4AP channel	8.06 nS [15]
The number of channels in cell	
funny channel (*nf*)	9600
L type Ca channel (*nLCa*)	5156
T type Ca channel (*nTCa*)	1011
Ks channel (*nKs*)	286
Kr channel (*nKr*)	895
Sustained channel (*nSus*)	1000
4AP channel (*n4AP*)	1000
ryanodine receptors in cell (*nrr*)	135000 [15]

Table S3. Mathematical equations of sinoatrial nodal cells

Membrane potential (V_m)

$dV_m/dt = -I_{tot}/C_m$

Membrane current (I_{tot})

$I_{tot} = I_{LCa} + I_{TCa} + I_{Kr} + I_{Ks} + I_{to} + I_{ss} + I_{fNa} + I_{fK} + I_{st} + I_{NaK} + I_{NCX} + I_{bNa} + I_{bCa}$

funny current ($I_f = I_{fNa} + I_{fK}$) [15,13]

yi = 1.0/(1.0+exp((V_m+64.0)/13.5))

Ty = 0.7166529/(exp(-(V_m+386.9)/45.302)+exp((V_m-73.08)/19.231))

$dy/dt = (ti - y) * Ty$

ENa = 26.7266*log(Na_I / Na_C)

EK = 26.7266*log(K_I / K_C)

$I_{fNa} = C_m$ * 0.3833*0.15* ($V_m - ENa$)*y^2

$I_{fK} = C_m$ * 0.6167*0.15* ($V_m - EK$)*y^2

$I_f = I_{fNa} + I_{fK}$

L-type Ca current (I_{LCa}) [15,13]

dLi = 1.0/(1.0+exp(-(V_m+13.5)/6.0))

adL = -0.02839*(V_m+35.0)/(exp(-(V_m+35.0)/2.5)-1.0) - 0.0849*V_m/(exp(-V_m/4.8)-1.0)

bdL = 0.01143*(V_m-5.0)/(exp(V_m-5.0)/2.5)-1.0)

TdL = 1.0/(adL + bdL)

$ddL/dt = (dLi - dL) * TdL$

fLi = 1.0/(1.0+exp((V_m+35.0)/7.3))

TfL = 257.1*exp(-((V_m+32.5)/13.9)^2)+44.3

$dfL/dt = (fLi - fL) * TfL$

$fCai$ = 0.00035/(0.00035+Ca_S)

$TfCa$ = $fCai$/0.021

$dfC/dt = (fCai - fC) * TfCa$

$I_{LCa} = C_m$ * 0.58 * (V_m – 45.0)*dL*fL*fC

T-type Ca current (I_{TCa}) [15,13]

dTi = 1.0/(1.0+exp(-(V_m+26.3)/6.0))

TdT = 1.0/(1.068*exp((V_m+26.3)/30.0)+1.068*exp(-(V_m+26.3)/30.0))

$ddT/dt = (dTi - dT) * TdT$

fTi = 1.0/(1.0+exp((V_m+61.7)/5.6))

TfT = 1.0/(0.0153*exp(-(V_m+61.7)/83.3)+0.015*exp((V_m+61.7)/15.38))

$dfT/dt = (fTi - fT) * TfT$

$I_{TCa} = C_m$ * 0.1832 * (V_m – 45.0)*dT*fT

Kr current (I_{Kr}) [15,13]

Pai = 1.0/(1.0+exp(-(V_m+23.2)/10.6))

$TPai$ = 0.8466/(0.0372*exp(V_m/15.9)+0.00096*exp(-V_m/22.5)

$dPaF/dt = (Pai - PaF) * TPai$

$TPaS$ = 0.8466/(0.0042*exp(V_m/17.0)+0.00015*exp(-V_m/21.6)

$dPaS/dt = (Pai - PaS) * TPaS$

Pii = 1.0/(1.0+exp((V_m+28.6)/17.1))

$TPii$ = 1.0/(0.1*exp(-V_m/54.645)+0.656*exp(V_m/106.157)

$dPi/dt = (Pii - Pi) * TPii$

EK = 26.7266*log(K_I/K_C)

$I_{Kr} = C_m$ * 0.08114 * (V_m – EK)*(0.6*PaF+0.4*PaS)*Pi

Ks current (I_{Ks}) [15,13]

Ksa = 0.014/(1.0+exp(-(V_m-40.0)/9.0))
Ksb = 0.001*exp(-V_m/45.0)
ni = Ksa/(Ksa+Ksb)
Tn = 1.0/(Ksa+Ksb)
$dn/dt = (ni - n) * Tn$
EKs = 26.7266*log((K_I +0.12*Na_I)/(K_C +0.12*Na_C))
$I_{Ks} = C_m$ * 0.0259 * (V_m – EKs)*n^2

Sustained inward current (I_{st}) [15,13]

qai = 1.0/(1.0+exp(-(V_m+57.0)/5.0))
aqa = 1.0/(0.15*exp(-V_m/11.0)+0.2*exp(-V_m/700.0))
bqa = 1.0/(16.0*exp(V_m/8.0)+15.0*exp(V_m/50.0))
Tqa = 1.0/(aqa+bqa)
$dqa/dt = (qai - qa) * Tqa$
aqi = 1.0/(3100.0*exp(V_m/13.0)+700.0*exp(V_m/70.0))
bqi = 1.0/(95.0*exp(-V_m/10.0)+50.0*exp(-V_m/700.0))+0.000229/(1.0+exp(-V_m/5.0))
qii = aqi / (aqi+bqi)
Tqi = 6.65 / (aqi+bqi)
$dqi/dt = (qii - qi) * Tqi$
$I_{st} = C_m$ * 0.003* (V_m – 37.4)*qa*qi

4-aminopyridine-sensitive current ($I_{4AP} = I_{to} + I_{ss}$) [15,13]

qi = 1.0/(1.0+exp((V_m+49.0)/13.0))
Tq = 6.06 + 39.102/(0.57*exp(-0.08*(V_m+44.0))+0.065*exp(0.1*(V_m+45.93)))
$dq/dt = (qi - q) * Tq$
ri = 1.0/(1.0+exp(-(V_m-19.3)/15.0))
Tr = 2.75352 + 14.40516/(1.037*exp(0.09*(V_m+30.61))+0.369*exp(-0.12*(V_m+23.84)))
$dr/dt = (ri - r) * Tr$
EK = 26.7266*log(K_I / K_C)
$I_{to} = C_m$ * 0.252* (V_m – EK)*q*r
$I_{ss} = C_m$ * 0.02* (V_m – EK)*r
$I_{4AP} = I_{to} + I_{ss}$

Na/Ca exchanger current (I_{NCX}) [15,13]

do = 1.0 + (Ca_I/3.663)*2.0)+ (Na_I /1628.0)*(1.0+(Na_I /561.4)*(1.0+ Na_I /4.663))
di = 1.0 + (Ca_S /0.0207)*(1.0+exp(-0.1369*V_m/26.72655)+ Na_C /26.44)
+ (Na_C /395.3)*(1.0+(NaS/2.289)*(1.0+ Na_C /26.44))
k_{12} = (Ca_S /0.0207)*exp(-0.1369*V_m/26.72655) / di;
k_{14} = (Na_C /395.2)*(Na_C /2.289)*(1.0+ Na_C /26.44)*exp(0.4315*V_m/(2*26.72655))/di;
k_{21} = (Ca_I/3.663) /do;
k_{23} = (Na_I /K1628.0)*(Na_I /561.4)*(1.0+ Na_I /4.663)*exp(-0.4315*V_m/(2*26.72655))/do;
k_{32} = exp(0.4315*V_m/(2*26.72655));
k_{34} = Na_I /(4.663+ Na_I);
k_{41} = exp(-0.4315*V_m/(2*26.72655));
k_{43} = Na_C /(26.44+ Na_C);
$x_1 = k_{34}*k_{41}*(k_{23}+k_{21}) + k_{21}*k_{32}*(k_{43}+k_{41})$;
$x_2 = k_{43}*k_{32}*(k_{14}+k_{12}) + k_{41}*k_{12}*(k_{34}+k_{32})$;
$x_3 = k_{43}*k_{14}*(k_{23}+k_{21}) + k_{12}*k_{23}*(k_{43}+k_{41})$;
$x_4 = k_{34}*k_{23}*(k_{14}+k_{12}) + k_{21}*k_{14}*(k_{34}+k_{32})$;
$I_{NCX} = C_m * 187.5 * (k_{21}*x_2-k_{12}*x_1)/(x_1+x_2+x_3+x_4)$;

Na/K pump current (I_{NaK}) [15,13]
$ENa = 26.7266*\log(Na_I / Na_C)$
$aNaK = 1.0/(1.0+(1.4/ K_I)^{1.2})$
$bNaK = 1.0/(1.0+(14.0/ Na_C)^{1.3})$
$cNaK = 1.0/(1.0+\exp(-(V_m-ENa+120.0)/30.0))$
$I_{NaK} = C_m * 2.88 * aNaK * bNaK * cNaK$

ryanodine receptors Ca^{2+} release flux (J_{rel})
$Y = 1.0 – 0.0005*exp(Ca_S /0.0002-1.0)-0.00001*exp(CaJ/0.5-1.0)$
for (i=1 ;i<=nrr ;i++){
if ($rE > Y$) {rP=0.5*Y; $drE/dt = (rP - rE)$; }
else if ($rE < 0.7$) { rP = rand(0,1) ; $rE = 0.95*Y$; }
else { $drP/dt = 30.0 * rP * (1.0 - rP) * (rP - Y)$; $drE/dt = 0.1 * (rP - rE)$; }
if ($rP > Y$) {*count*++ ;} }
RyR = *count* / nrr ;
$J_{rel} = count*0.60*(CaJ- Ca_S)/0.6/(2.0*F*V_{suv})$

SERCa pump Ca^{2+} uptake flux from cytosol to NSR (J_{sru})
$J_{sru} = 0.012*(Ca_C /0.000139-CaN/1.706052)/(1.0+ Ca_C /0.000139+CaN/1.706052)$

Na^+ background current (I_{bNa}) [15,13] $ENa =$
$26.7266*\log(Na_I / Na_C)$
$I_{bNa} = C_m * 0.00486 *(V_m-ENa)$

Ca^{2+} background current (I_{bCa}) [15,13]
$I_{bNa} = C_m * 0.0006 *(V_m-45.0)$

Ca^{2+} transfer rate from subspace to cytosol (J_{diff}) [15,13]
$J_{diff} = (Ca_S - Ca_C)/0.04$

Ca^{2+} transfer rate from NSR to JSR (J_{trd}) [15,13]
$J_{trd} = (CaN - CaJ)/40.0$

Ca^{2+} buffering : Fraction of Calmodulin and Ca^{2+} complex in cytosol (*fCMi*) [15,13]
$dfCMi/dt = 227.7 * CaC * (1.0 - fCMi) - 0.542 * fCMi$

Ca^{2+} buffering : Fraction of Calmodulin and Ca^{2+} complex in subspace (*fCMs*) [15,13]
$dfCMs/dt = 227.7 * CaS * (1.0 - fCMs) - 0.542 * fCMs$

Ca^{2+} buffering : Fraction of Toponin_Ca and Ca^{2+} complex in cytosol (*fTC*) [15,13]
$dfTC/dt = 88.8 * CaC * (1.0 - fTC) - 0.446 * fTC$

Ca^{2+} buffering : Fraction of Toponin_Mg and Ca^{2+} complex in cytosol (*fTMC*) [15,13]
$dfTMC/dt = 227.7 * CaC * (1.0 - fTMC) - 0.00751 * fTMC$

Ca^{2+} buffering : Fraction of Toponin_Mg and Mg^{2+} complex in cytosol (*fTMM*) [15,13]
$dfTMM/dt = 2.277 * MgC * (1.0 - fTMM) - 0.751 * fTMM$

Ca^{2+} buffering : Fraction of Calsequestirn and Ca^{2+} complex in JSR (*fCQ*) [15,13]

$dfCQ/dt = 0.534 * CaJ * (1.0 - fCQ) - 0.445 * fCQ$

Mass ballance: K^+ concentration in cytosol (K_C)

$$dKC/dt = -(I_{Kr} + I_{Ks} + I_{to} + I_{SS} + I_{fK} - 2 * I_{NaK})/(F * V_c)$$

Mass ballance: Ca^{2+} concentration in cytosol (Ca_C)

$$dCaC/dt = \left(J_{diff} * V_{sub} - J_{sru} * V_{NSR}\right)/V_{cyt}$$
$$-(CM_{tot} * fCMi + TC_{tot} * fTC + TMC_{tot} * fTMC)$$

Mass ballance: Ca^{2+} concentration in subspace (Ca_S)

$$dCaS/dt = J_{rel} * V_{jsr}/V_{sub}$$
$$-(I_{LCa} + I_{TCa} + I_{bCa} - 2 * I_{NCX})/(2 * F * V_{sub}) - (J_{diff} + CM_{tot} * fCMs)$$

Mass ballance: Ca^{2+} concentration in NSR (CaN)

$$dCaN/dt = J_{sru} - J_{trd} * V_{jsr}/V_{NSR}$$

Mass ballance: Ca^{2+} concentration in JSR (CaJ)

$$dCaJ/dt = J_{trd} - J_{rel} - CQ_{tot} * fCQ$$

Mass ballance: Na^+ concentration in cytosol (Na_C)

$$dNaC/dt = -(I_{fNa} + I_{st} + I_{bNa} + 3 * I_{NCX} + 3 * I_{NaK})/(F * V_c)$$

Supplementary information Figure S1 illustrates four divided intracellular spaces (subspace, cytosol, the network sarcoplasmic reticulum, and the junctional sarcoplasmic reticulum) and an arrangement of the more than 10 types of ion channel, the pumps, and the exchanger.

Table S4. Patient physical properties and treatment conditions

Total body fluid volume	38.40 L
Plasma fluid volume (V_P)	2.16 L
Interstitial fluid volume (V_I)	11.04 L
Intracellular fluid volume (V_C)	25.20 L
Ultrafiltration volume	10.0 mL/min
Plasma refiling rate	10.0 mL/min
Blood flow rate in dialyzer	200.0 mL/min
Dialysis fluid flow rate in dialyzer	500.0 mL/min
Hematocrit	35 %
Treatment time	4.0 hour
The number of treatments	3 times / week

[13] Kurata, Y.; Hisatome, I.; Imanishi, S.; Shibamoto, T. Dynamical description of sinoatrial node pacemaking: improved math-ematical model for primary pacemaker cell. *Am. J. Physiol. Heart. Circ. Physiol.* **2002**, *283*, H2074–101.
[14] Wilders, R. Computer modelling of the sinoatrial node. *Med. Biol. Eng. Comput.* **2007**, *45*, 189–207.
[15] Maltsev, V.A.; Lakatta, E.G. A novel quantitative explanation for the autonomic modulation of cardiac pacemaker cell auto-maticity via a dynamic system of sarcolemmal and intracellular proteins. Am. J. Physiol. Heart. Circ. Physiol. 2010, 298, H2010–23.
